# Supplementary material for: Essential role of TMPRSS2 in SARS-CoV-2 infection in murine airways
Source: Nat Commun. 2022 Oct 15;13:6100. doi: 10.1038/s41467-022-33911-8 (PMC9568946; doi:10.1038/s41467-022-33911-8)
Supplement: Supplementary file 1 — Supplementary Information [file 41467_2022_33911_MOESM1_ESM.pdf]

Supplementary Information

Essential role of TMPRSS2 in SARS-CoV-2 infection in murine airways

Naoko Iwata-Yoshikawa[1,7], Masatoshi Kakizaki[2,7], Nozomi Shiwa-Sudo[1], Takashi Okura[2], Maino Tahara[2], Shuetsu Fukushi[3], Ken Maeda[4], Miyuki Kawase[2], Hideki Asanuma[5], Yuriko Tomita[5], Ikuyo Takayama[5], Shutoku Matsuyama[5], Kazuya Shirato[2], Tadaki Suzuki[1], Noriyo Nagata[1]*, Makoto Takeda[2, 6]*

[1]Department of Pathology, National Institute of Infectious Diseases, Tokyo, Japan.
[2]Department of Virology III, National Institute of Infectious Diseases, Tokyo, Japan.
[3]Department of Virology I, National Institute of Infectious Diseases, Tokyo, Japan.
[4]Department of Veterinary Science, National Institute of Infectious Diseases, Tokyo, Japan.
[5]Center for Influenza and Respiratory Virus Research
[6]Department of Microbiology, Graduate School of Medicine and Faculty of Medicine, The University of Tokyo, Japan

[7]These authors contributed equally to this work: Naoko Iwata-Yoshikawa, Masatoshi Kakizaki
*Address correspondence to Noriyo Nagata, nnagata@niid.go.jp and Makoto Takeda, mtakeda@m.u-tokyo.ac.jp

Supplementary Table S1
Supplementary Figures S1–S4

Supplementary Table 1

Name	Simplified name	WHO label	Panglin lineage	GISAID accession no.
hCoV-19/Japan/TY-WK-521/2020	WK-521	none	A	EPI_ISL_408667
hCoV-19/Japan/QHN001/2020	QHN001	Alpha	B.1.1.7	EPI_ISL_804007
hCoV-19/Japan/QK002/2020	QK002	Alpha	B.1.1.7	EPI_ISL_768526
hCoV-19/Japan/QHN002/2020	QHN002	Alpha	B.1.1.7	EPI_ISL_804008
hCoV-19/Japan/TY8-612-P1/2021	TY8-612	Beta	B.1.351	EPI_ISL_1123289
hCoV-19/Japan/TY8-689-P0/2021	TY8-689	Beta	B.1.351	EPI_ISL_2158605
hCoV-19/Japan/TY7-501/2021	TY7-501	Gamma	P.1	EPI_ISL_833366
hCoV-19/Japan/TY7-503/2021	TY7-503	Gamma	P.1	EPI_ISL_877769
hCoV-19/Japan/TY8-886-P1/2021	TY8-886	Gamma	P.1	EPI_ISL_1358216
hCoV-19/Japan/TY11-927-P1/2021	TY11-927	Delta	AY.122	EPI_ISL_2158617
hCoV-19/Japan/TY11-330-P1/2021	TY11-330	Kappa	B.1.617.1	EPI_ISL_2158613
hCoV-19/Japan/TY38-873P0/2021	TY38-873	Omicron	BA.1	EPI_ISL_7418017
hCoV-19/Japan/NC928-2N/2021	NC928	Omicron	BA.1	EPI_ISL_7507055
hCoV-19/Japan/TY40-730-P0/2022	TY40-730	Omicron	BA.1	EPI_ISL_9595827
hCoV-19/Japan/TY40-733-P0/2022	TY40-733	Omicron	BA.1.1	EPI_ISL_9595828
hCoV-19/Japan/TY40-737-P0/2022	TY40-737	Omicron	BA.1.1	EPI_ISL_9595829
hCoV-19/Japan/TY40-779-P0/2022	TY40-779	Omicron	BA.1	EPI_ISL_9595836
hCoV-19/Japan/TY40-395-P0/2022	TY40-395	Omicron	BA.1.1	EPI_ISL_9596136
hCoV-19/Japan/TY40-412-P0/2022	TY40-412	Omicron	BA.1	EPI_ISL_9595821
hCoV-19/Japan/TY40-449-P0/2022	TY40-449	Omicron	BA.1	EPI_ISL_9595824
hCoV-19/Japan/TY40-467	TY40-467	Omicron	BA.1.1	Not registered
QHmusX (mouse passaged hCoV-19/Japan/QH-329-037/2020)	QHmusX	none	B.1	None (GenBank accession LC605054)

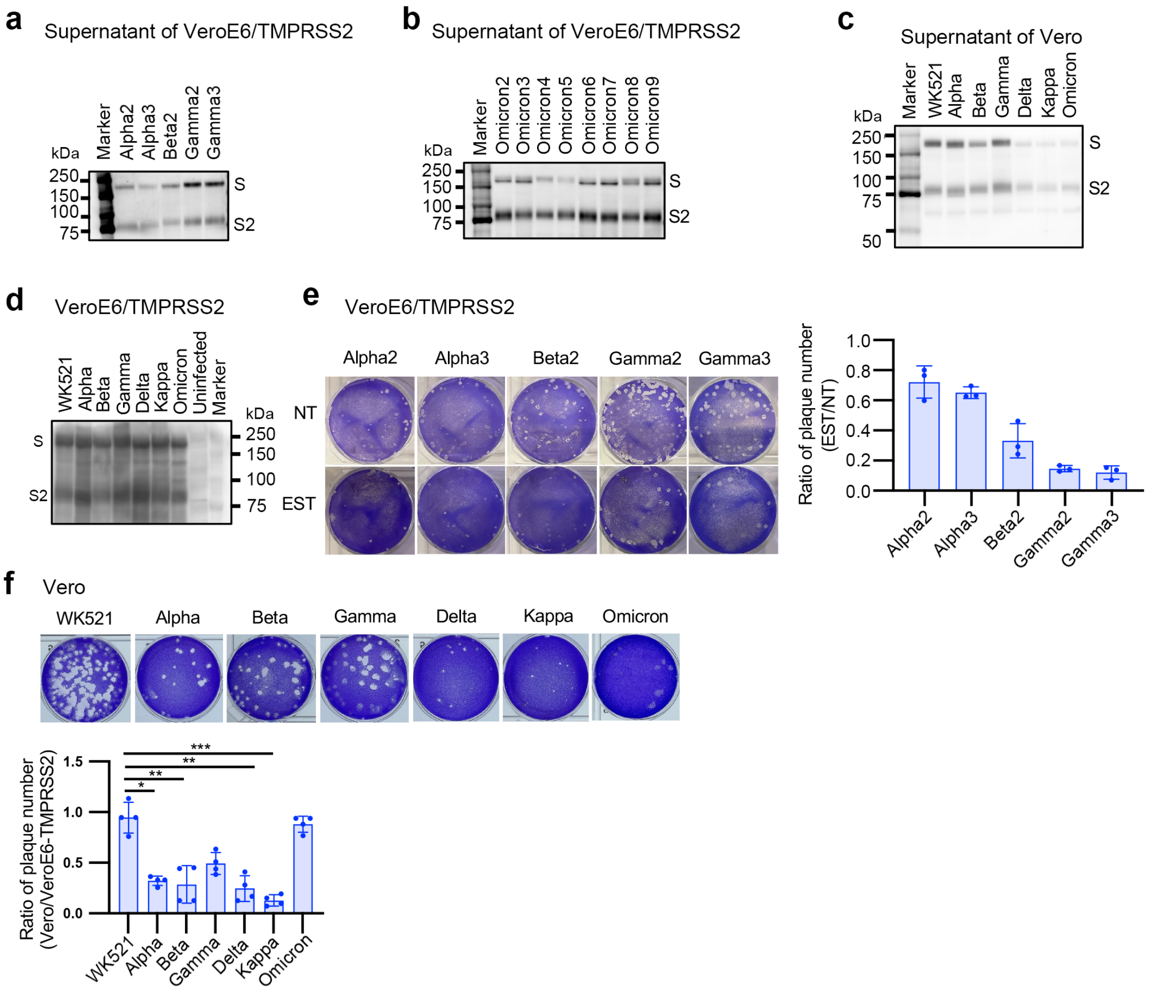

Supplementary Fig. 1. Spike protein cleavage and entry phenotype of SARS-CoV-2 in VeroE6/TMPRSS2 and Vero cells. (**a, b**) S proteins detected in the culture supernatant of infected VeroE6/TMPRSS2 cells. SDS-PAGE and western blot analysis. Data are representative of two independent experiments. (**c**) S proteins detected in the culture supernatant of infected Vero cells. SDS-PAGE and western blot analysis. Data are representative of two independent experiments. (**d**) S proteins detected in the infected VeroE6/TMPRSS2 cells. SDS-PAGE and western blot analysis. Data are representative of two independent experiments. (**e**) Plaque formation on VeroE6/TMPRSS2 cells in the absence (not treated: NT) or in the presence of EST. The bar graph shows the ratio of plaque number in EST-treated cells to that in untreated cells (NT). Error bars indicate the standard deviations (SD) of triplicate wells. Mean values ± SD are shown. (**f**) Plaque

formation on Vero cells. For infection, the same infectious titre of virus was used in the experiment with VeroE6/TMPRSS2 cells in Fig. 1d. The bar graph shows the ratio of plaque number in Vero cells to that in VeroE6/TMPRSS2 cells. Error bars indicate the standard deviations of quadruplicate wells. Mean values ± SD are shown, and significant differences were determined with Kruskal-Wallis test. Multiple comparisons between WK521 and other variants were adjusted with uncorrected Dunn's multiple comparison test, *P=0.0143 (vs Alpha), **P=0.0099 (vs Beta), **P=0.0052 (vs Delta), and ***P=0.0003 (vs Kappa). Alpha (QHN001), Alpha2 (QK002), Alpha3 (QHN002), Beta (TY8-612), Beta2 (TY8-689), Gamma (TY7-501), Gamma2 (TY7-503), Gamma3 (TY8-886), Delta (TY11-927), Kappa (TY11-330), Omicron (TY38-873), Omicron2 (TY40-412), Omicron3 (TY40-395), Omicron4 (TY40-449), Omicon5 (TY40-467), Omicron7 (TY40-733), Omicron8 (TY40-737), Omicron9 (TY40-779).

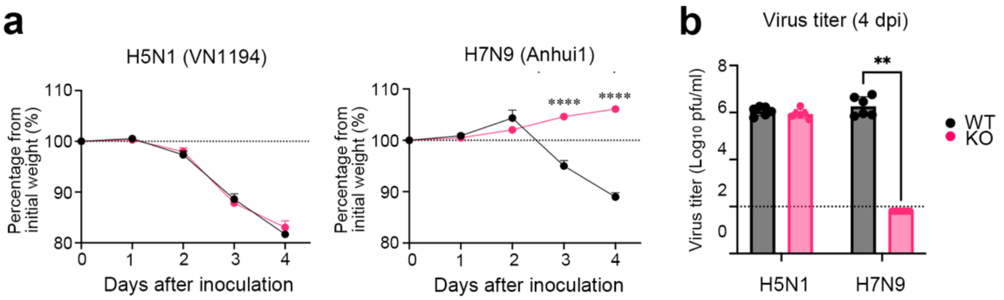

Supplementary Fig. 2. Experimental infection of TMPRSS2-knockout (KO) mice with influenza virus H5N1 and H7N9 subtype strains. C57BL/6 (WT) and TMPRSS2-KO mice were intranasally inoculated with 10^4 PFU of H7N9 (Anhui1) or H5N1 (VN1194). (**a**) The body weights were measured daily (n = 6 per group, 3 males and 3 females). ****P < 0.0001 by two-way ANOVA followed by Bonferroni's multiple comparison test. Data are mean ± SEM. (**b**) Bronchoalveolar lavage fluid at 4 dpi was used for viral titration (n = 6 per group, 3 males and 3 females). The detection limit was 10^2 PFU/mL of bronchoalveolar lavage fluid. **P = 0.0022 by two-tailed Mann–Whitney U test. Data are mean ± SD.

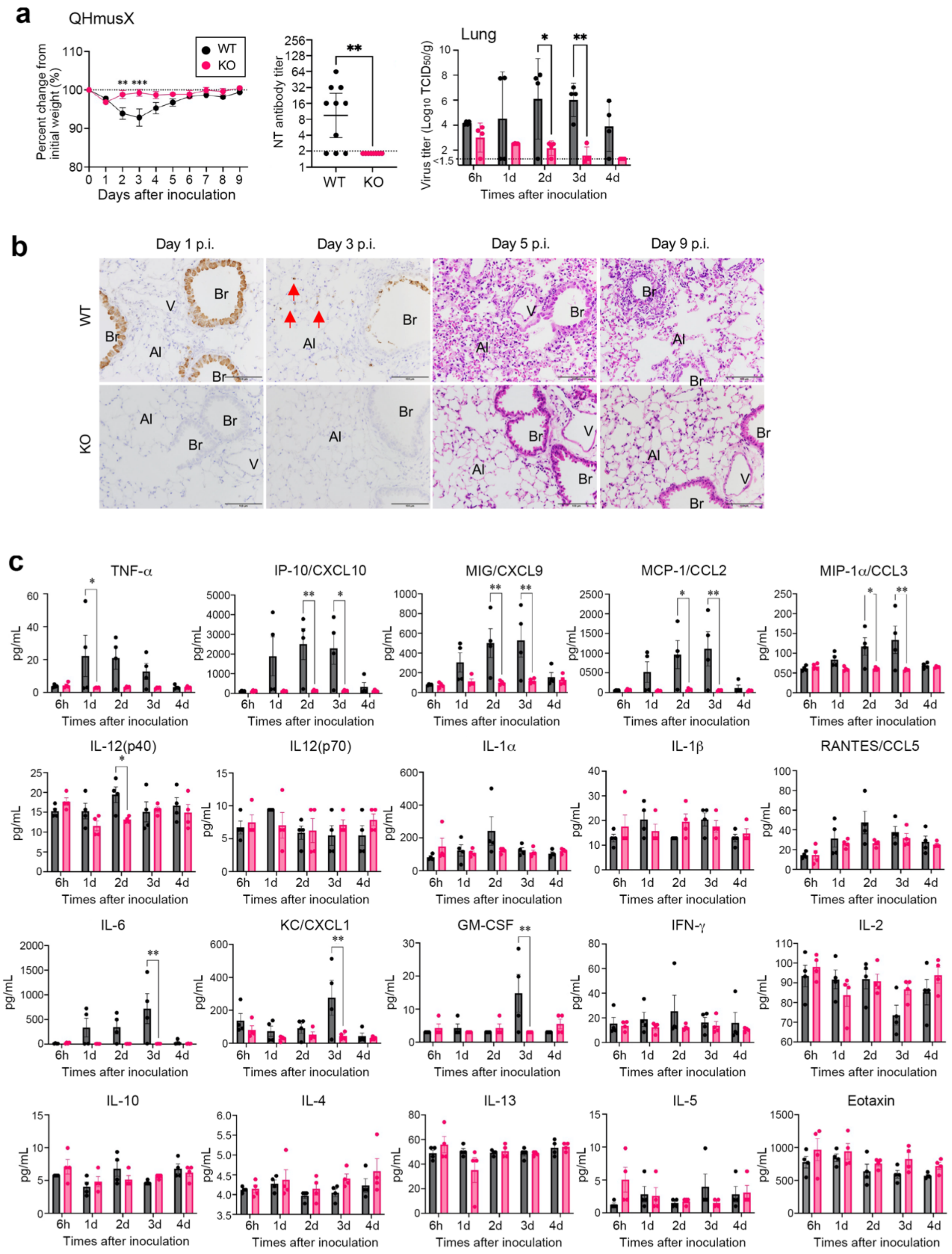
a
QHmusX
WT
KO
Percent change from initial weight (%)
Days after inocularion
NT antibody titer
Lung
Virus titer (Log10 TCID50/g)
Times after inoculation
b
Day 1 p.i.
Day 3 p.i.
Day 5 p.i.
Day 9 p.i.
WT
KO
c
TNF-α
IP-10/CXCL10
MIG/CXCL9
MCP-1/CCL2
MIP-1α/CCL3
IL-12(p40)
IL12(p70)
IL-1α
IL-1β
RANTES/CCL5
IL-6
KC/CXCL1
GM-CSF
IFN-γ
IL-2
IL-10
IL-4
IL-13
IL-5
Eotaxin
pg/mL
Times after inoculation

Supplementary Fig. 3. Experimental infection of TMPRSS2-knockout (KO) mice with a mouse-adapted SARS-CoV-2 strain. C57BL/6 (WT) and TMPRSS2-KO mice were inoculated intranasally with 1.9×10^4 $TCID_{50}$ of strain QHmusX. (**a** left panel) Body weight curve during the observation period. Asterisks indicate statistically significant differences between mice (n = 8 (KO, 4 males and 4 females), n = 10 (WT, 5 males and 5 females); **P = 0.0069 and ***P = 0.0001 by two-way ANOVA followed by Bonferroni's multiple comparison test). Data are mean ± SEM. (**a** middle panel) Serum neutralization titres (NT) against strain QHmusX at 9 days post-infection (p.i.). The dashed line indicates the limit of detection (< 2) (n = 8 (KO, 4 males and 4 females), n = 10 (WT, 5 males and 5 females); **P = 0.0053 by two-tailed Mann–Whitney U test). Data with a geometric mean (GMT) + 95% confidence interval, CI. (**a** right panel) Viral titres in lung homogenates at 6 h and 1 to 4 days p.i. (n = 4 per group, 2 males and 2 females). The detection limit was $10^{1.5}$ $TCID_{50}$/g of tissue. *P = 0.0203 and **P = 0.0072 by two-way ANOVA followed by Bonferroni's multiple comparison test. Data are mean ± SD. (**b**) Histopathology of the lungs from WT and TMPRSS2-KO mice at 1, 3, 5, and 9 days p.i. (n = 4 per group, 2 males and 2 females). Immunohistochemical analysis using an anti-SARS-CoV-2 NP antibody (at 1 and 3 days p.i.). Red arrows indicate representative viral antigen-positive cells in alveoli. Haematoxylin and eosin staining at 5 and 9 days p.i. Br, bronchi; V, vein. Bars, 100 μm. (**c**) Cytokine and chemokine levels in the lungs of mice at different time points after inoculation (n = 4 per group, 2 males and 2 females). Samples were from the same experiment as those in C. *P = 0.0386 (TNF-α), 0.0176 (IP-10/CXCL10), 0.0174 (MCP-1/CCL2), 0.0283 (MIP-1α/CCL3), and 0.0494 (IL-12 (p40)), **P = 0.0076 (IP-10/CXCL10), 0.0061 (MIG/CXCL9, 2 dpi), 0.0051 (MIG/CXCL9, 3 dpi), 0.0035 (MCP-1/CCL2), 0.0019 (MIP-1α/CCL3), 0.0014 (IL-6), 0.0017 (KC/CXCL1), and 0.0011 (GM-CSF) by two-way ANOVA followed by Bonferroni's multiple comparison test. Data are mean ± SEM. (**c**): Each dot represents data from an individual animal (n = 4).

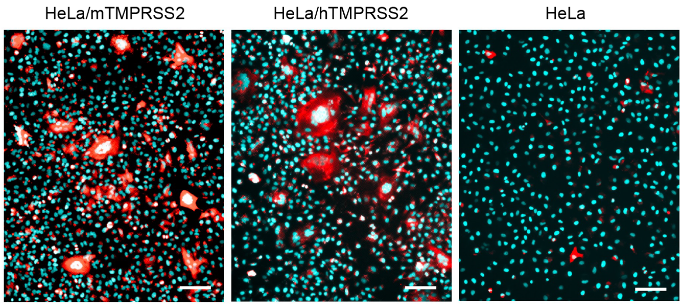

Supplementary Fig. 4. Syncytium formation induced by TMPRSS2 expression. Syncytium formation in HeLa cells expressing mouse and human TMPRSS2 (HeLa/mTMPRSS2 and HeLa/hTMPRSS2, respectively) and the parental HeLa cells. Cells were transfected with the Omicron S protein expression plasmid together with human ACE2-expression plasmid. At 2 days post-transfection, S protein was detected by an indirect immunofluorescent assay (red) and nuclei were detected by DAPI staining (blue). Scale bars indicate 100µm.
