# Supplementary material for: Essential role of TMPRSS2 in SARS-CoV-2 infection in murine airways
Source: Nat Commun. 2022 Oct 15;13:6100. doi: 10.1038/s41467-022-33911-8 (PMC9568946; doi:10.1038/s41467-022-33911-8)
Supplement: Supplementary file 3 — Reporting Summary [file 41467_2022_33911_MOESM3_ESM.pdf]

nature portfolio

Corresponding author(s): Makoto Takeda

Last updated by author(s): Sep 26, 2022

Reporting Summary

Nature Portfolio wishes to improve the reproducibility of the work that we publish. This form provides structure for consistency and transparency in reporting. For further information on Nature Portfolio policies, see our Editorial Policies and the Editorial Policy Checklist.

Statistics

For all statistical analyses, confirm that the following items are present in the figure legend, table legend, main text, or Methods section.

n/a	Confirmed	
☐	☒	The exact sample size (*n*) for each experimental group/condition, given as a discrete number and unit of measurement
☐	☒	A statement on whether measurements were taken from distinct samples or whether the same sample was measured repeatedly
☐	☒	The statistical test(s) used AND whether they are one- or two-sided<br>*Only common tests should be described solely by name; describe more complex techniques in the Methods section.*
☒	☐	A description of all covariates tested
☒	☐	A description of any assumptions or corrections, such as tests of normality and adjustment for multiple comparisons
☐	☒	A full description of the statistical parameters including central tendency (e.g. means) or other basic estimates (e.g. regression coefficient) AND variation (e.g. standard deviation) or associated estimates of uncertainty (e.g. confidence intervals)
☐	☒	For null hypothesis testing, the test statistic (e.g. *F*, *t*, *r*) with confidence intervals, effect sizes, degrees of freedom and *P* value noted<br>*Give P values as exact values whenever suitable.*
☒	☐	For Bayesian analysis, information on the choice of priors and Markov chain Monte Carlo settings
☒	☐	For hierarchical and complex designs, identification of the appropriate level for tests and full reporting of outcomes
☒	☐	Estimates of effect sizes (e.g. Cohen's *d*, Pearson's *r*), indicating how they were calculated

Our web collection on statistics for biologists contains articles on many of the points above.

Software and code

Policy information about availability of computer code

Data collection	Luminex xPONENT (version 4.2), Thermo Fisher Scientific, http://thermofisher.com<br>LightCycler® 480 Software (version 1.5.1), Roche Molecular Systems, Inc., https://lifescience.roche.com/<br>OLYMPUS cellSense Standard 2.1, www.olympus-sis.com<br>Amersham Imager 800 (Cytiva)<br>All-in-One Fluorescence Microscope BZ-X800 (Keyence)
Data analysis	GraphPad Prism 9 (version 9.4.1), GraphPad Software, https://www.graphpad.com/<br>Fiji software v2.2.0 (ImageJ)

For manuscripts utilizing custom algorithms or software that are central to the research but not yet described in published literature, software must be made available to editors and reviewers. We strongly encourage code deposition in a community repository (e.g. GitHub). See the Nature Portfolio guidelines for submitting code & software for further information.

Data

Policy information about availability of data

All manuscripts must include a data availability statement. This statement should provide the following information, where applicable:

- Accession codes, unique identifiers, or web links for publicly available datasets
- A description of any restrictions on data availability
- For clinical datasets or third party data, please ensure that the statement adheres to our policy

All data supporting the findings of this study are available within the paper and in the Source Data. There are no restrictions in obtaining access to primary data. All unique/stable reagents generated in this study are available from the Lead Contact upon furnishing a completed Materials Transfer Agreement. The sequences of the virus isolates used are available in the Global Initiative on Sharing All Influenza Data (GISAID) database. The list of virus names used in this study and GISAID ID numbers are shown in Supplementary Table 1. The cell line information of VeroE6/TMPRSS2 is available from JCRB Cell Bank in Japan (https://cellbank.nibiohn.go.jp/english/) (JCRB no. JCRB1819).

Human research participants

Policy information about studies involving human research participants and Sex and Gender in Research.

Reporting on sex and gender	Not applicable
Population characteristics	Not applicable
Recruitment	Not applicable
Ethics oversight	Not applicable

Note that full information on the approval of the study protocol must also be provided in the manuscript.

Field-specific reporting

Please select the one below that is the best fit for your research. If you are not sure, read the appropriate sections before making your selection.

☒ Life sciences ☐ Behavioural & social sciences ☐ Ecological, evolutionary & environmental sciences

For a reference copy of the document with all sections, see nature.com/documents/nr-reporting-summary-flat.pdf

Life sciences study design

All studies must disclose on these points even when the disclosure is negative.

Sample size	The sample sizes for the mouse studies were chosen because they have previously been shown to be sufficient to evaluate a significant difference among groups (Iwata-Yoshikawa and Shiwa et al. Science Advances, 2022; PMID 34995117). The sample size (n > 3) for cell culture experiments were chosen for applying statistical tests, because they have previously been shown to be sufficient to evaluate a significant difference among groups (Saito A, et al. Nature, 2021. Suzuki R, et al. Nature, 2022).
Data exclusions	No data was excluded.
Replication	In vitro experiments representative of at least 2 experiments with multiple samples per time point. Animal experiments were conducted independently in multiple biological replicates for each variant virus. All experiments with multiple biological replicates are indicated in the figure legends. Animal experiments were performed once unless otherwise stated. Samples from mice for viral genome copies, infectious viral titers, and histological examination were assayed in multiple biological replicates. All replication attempts were successful.
Randomization	For animal studies, mice were randomly assigned to groups in an age and sex-matched distribution. All experiments were derived from animal work so no additional randomization was required in downstream analysis. For experiments other than animal studies, randomization is not applicable because homogenous materials (i.e., cell line) were used.
Blinding	For the measurements of viral genome copies, infectious viral titers, and the histological examinations were blinded to group allocation to ensure the assessment was unbiased. For the in vitro and in vivo experiments, blinding was not possible because researchers were involved in infection/treatment procedures.

Reporting for specific materials, systems and methods

We require information from authors about some types of materials, experimental systems and methods used in many studies. Here, indicate whether each material, system or method listed is relevant to your study. If you are not sure if a list item applies to your research, read the appropriate section before selecting a response.

Materials & experimental systems

n/a	Involved in the study
☐	☒ Antibodies
☐	☒ Eukaryotic cell lines
☒	☐ Palaeontology and archaeology
☐	☒ Animals and other organisms
☒	☐ Clinical data
☒	☐ Dual use research of concern

Methods

n/a	Involved in the study
☒	☐ ChIP-seq
☒	☐ Flow cytometry
☒	☐ MRI-based neuroimaging

Antibodies

Antibodies used	IHC:<br>rabbit anti-SARS-CoV-2 N polyclonal antibody (in-house by Dr. Noriyo Nagata at the National Institute of Infectious Diseases; 1:5000; Iwata-Yoshikawa and Shiwa et al., Science Advances, 2022, PMID34995117).<br>Western blot:<br>rabbit anti-SARS-CoV-2 N polyclonal antibody (in-house by Dr. Noriyo Nagata at the National Institute of Infectious Diseases; 1:5000; Iwata-Yoshikawa and Shiwa et al., Science Advances, 2022, PMID34995117).<br>mouse anti-SARS-CoV-2 S1 subunit monoclonal antibody (clone # 1035206, R&D systems, Cat# MAB105403, 1:500)<br>rabbit anti-SARS-CoV-2 S2 subunit polyclonal antibody (Abcam, Cat# ab272504, 1:1000)<br>horseradish peroxidase (HRP)-conjugated F(ab')2 fragment of affinity purified anti-mouse IgG [H&L] [Goat] antibody (ROCKLAND, Cat#710-1332, 1:5000)<br>HRP-conjugated goat anti-rabbit IgG polyclonal antibody (MP Biomedicals, Cat# 55689, 1:5000).<br>For immunofluorescence staining:<br>rabbit anti-SARS-CoV-2 S polyclonal antibody ((Proteintech, Rosemont, Cat#28867-1-AP, 1:1,000)<br>Alexa 549-conjugated anti-rabbit IgG antibody (Thermo Fisher Scientific, Cat# A-11012, 1:2,000)
Validation	Validation of all primary antibodies for the species and application was conducted by manufacturers prior to sale, and validation statements are available on the manufacturers' website. Rabbit anti-SARS-CoV-2 N polyclonal antibody used in this study has been validated in previous study in Iwata-Yoshikawa and Shiwa et al., Science Advances, 2022, PMID34995117. mouse anti-SARS-CoV-2 S1 subunit monoclonal antibody (clone #1035206, R&D systems, Cat# MAB105403), rabbit anti-SARS-CoV-2 S2 subunit polyclonal antibody (Abcam, Cat# ab272504), horseradish peroxidase (HRP)-conjugated F(ab')2 fragment of affinity purified anti-mouse IgG [H&L] [Goat] antibody (ROCKLAND, Cat#710-1332), HRP-conjugated goat anti-rabbit IgG polyclonal antibody (MP Biomedicals, Cat# 55689), rabbit anti-SARS-CoV-2 S polyclonal antibody (Proteintech, Cat#28867-1AP), and Alexa 594-conjugated anti-rabbit IgG antibody (Thermo Fisher Scientific, Cat#A-11012) has been confirmed to give similar results as that described in publications by other groups (R&D systems, Cat# MAB105403: Bo Meng, et al. Cell Reports, 2022, PMID35963244. Abcam, Cat# ab272504: Qian Zhang, et al. Theranostics, 2021, PMID34335974. ROCKLAND, Cat#710-1332: Yuko Nariai, et al. Archives of Biochemistry and Biophysics, 2019, PMID30615852. MP Biomedicals, Cat# 55689: Jacqueline Ferralli, et al. Biology Open, 2018, PMID29555638. Proteintech, Cat#28867-1AP: Xiaojuan Zhou, et al. Cell Reports, 2021, PMID33932326. Thermo Fisher Scientific, Cat#A-11012: Xinyun Chen, et al. Nature Communications, 2021, PMID33397958). The staining patterns were checked for reproducibility and integrity of the assay in independent staining experiments and in positive samples.

Eukaryotic cell lines

Policy information about cell lines and Sex and Gender in Research

Cell line source(s)	A canine kidney cell line; MDCK cell line was obtained from Dr. Hideki Asanuma at the National Institute of Infectious Diseases (originally obtained from Dr, Peter Palese in the Department of Microbiology, Icahn School of Medicine at Mount Sinai.).<br>Vero cells [an African green monkey (Chlorocebus sabaeus) kidney cell line; JCRB0111]<br>VeroE6/TMPRSS2 cells (JCRB1819)<br>Calu-3 cells (a human lung epithelial cell line; ATCC HTB-55)<br>HeLa- hTMPRSS2 cells [HeLa229 cells (JCRB9086) stably expressing human TMPRSS2; Kawase et al., Journal of Virology, 2012]<br>HeLa- mTMPRSS2 cells [HeLa229 cells (JCRB9086) stably expressing mouse TMPRSS2; Kawase et al., Journal of Virology, 2012]
Authentication	None of the cells used were authenticated.
Mycoplasma contamination	All cell lines were tested for mycoplasma contamination and were confirmed to be mycoplasma-free.
Commonly misidentified lines<br>(See ICLAC register)	No cell lines were commonly misidentified.

Animals and other research organisms

Policy information about studies involving animals; ARRIVE guidelines recommended for reporting animal research, and Sex and Gender in Research

Laboratory animals	C57BL/6 mouse (male and female, 16 or 24 weeks old) were purchased from Japan SLC Inc. (Shizuoka, Japan). TMPRSS2 knockout line (male and female,14-34 weeks old) were established as previously described (Sakai et al., Journal of virology, 2014, PMID: 24600012) Mice were housed in groups of 5 to 6. Photoperiod = 12 hr on:12 hr off dark/light cycle. Ambient animal room temperature is 24 °C, and room humidity is 40-60%.
Wild animals	No wild animal was used.
Reporting on sex	Male and female animals were used in some of the animal studies in this study, but were not analyzed by sex because they showed similar susceptibility.
Field-collected samples	No field-collected samples were used.
Ethics oversight	Experiments using recombinant DNA and pathogens were approved by the Committee for Experiments using Recombinant DNA and Pathogens at the National Institute of Infectious Diseases, Tokyo, Japan (approval no. 2-84). All animal experiments were approved by the Animal Care and Use Committee of the National Institute of Infectious Diseases in Japan (approval no. 120153-II, 122045-II). All experimental animals were handled in BSL3 animal facilities according to the guidelines of this committee (approval no. 21-84, 22-53). All animals were housed in a facility certified by the Japan Health Sciences Foundation.

Note that full information on the approval of the study protocol must also be provided in the manuscript.
