# Supplementary material for: Essential role of TMPRSS2 in SARS-CoV-2 infection in murine airways
Source: Nat Commun. 2022 Oct 15;13:6100. doi: 10.1038/s41467-022-33911-8 (PMC9568946; doi:10.1038/s41467-022-33911-8)
Supplement: Supplementary file 4 — Source Data [file 41467_2022_33911_MOESM4_ESM.zip › 364185_2_related_ms_6934041_rkv6z2.pdf]

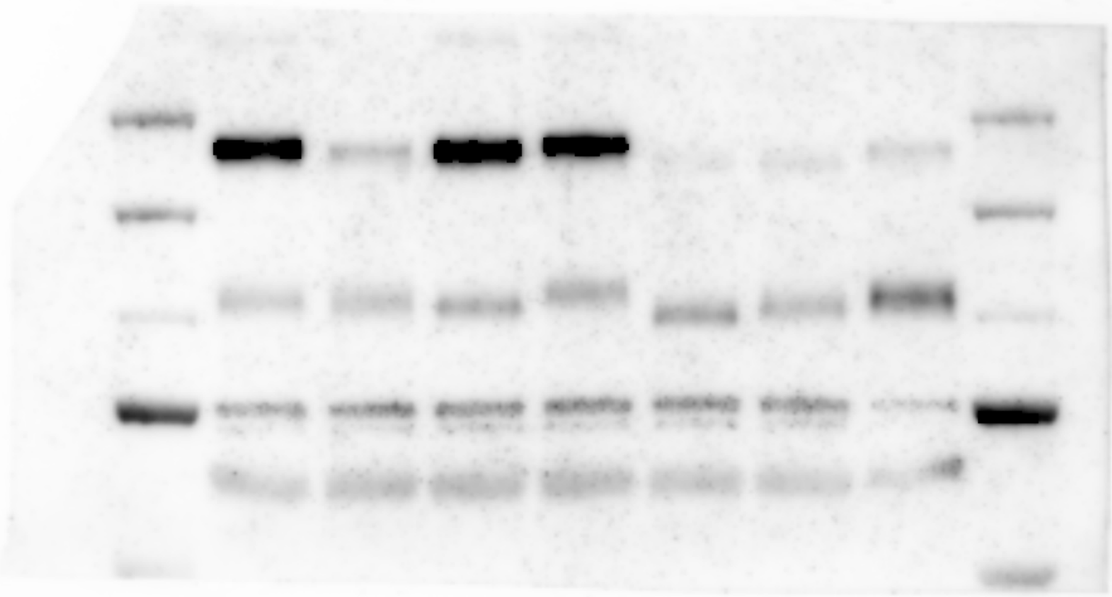

Fig. 1B Anti-S1

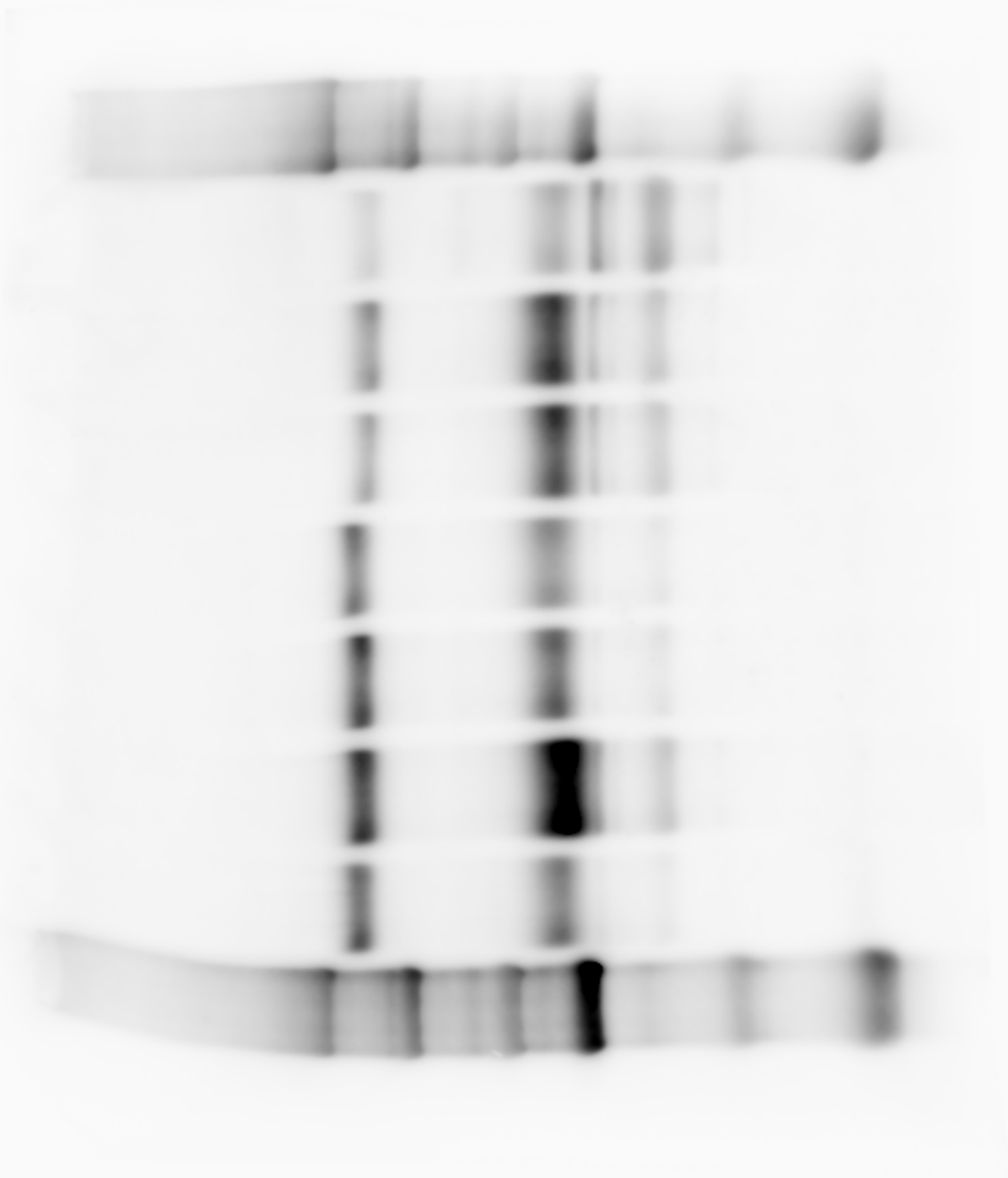

Fig. 1B Anti-S2

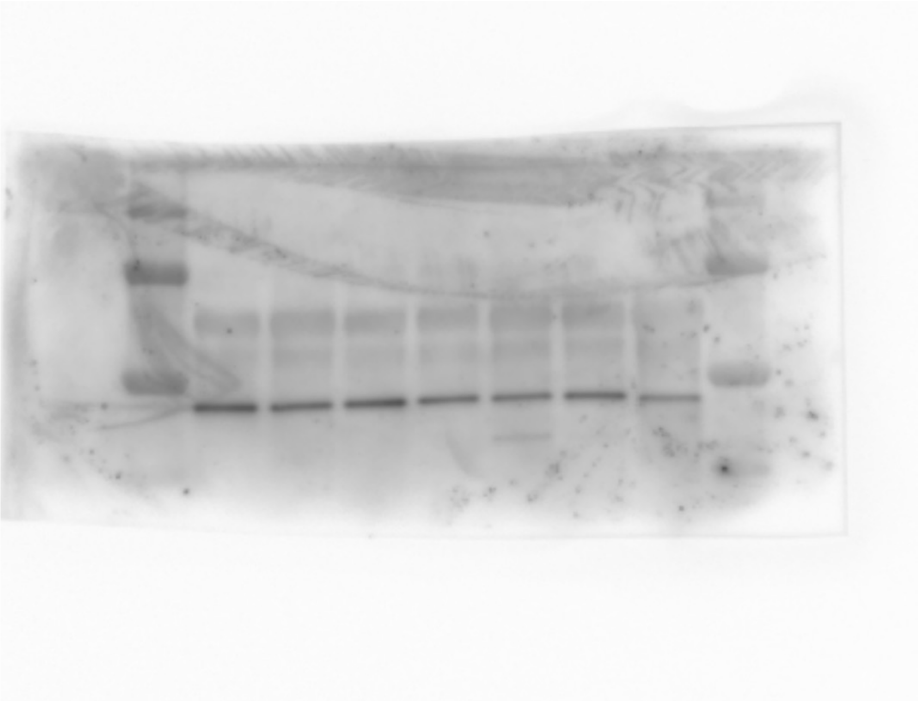

Fig. 1B Anti-N

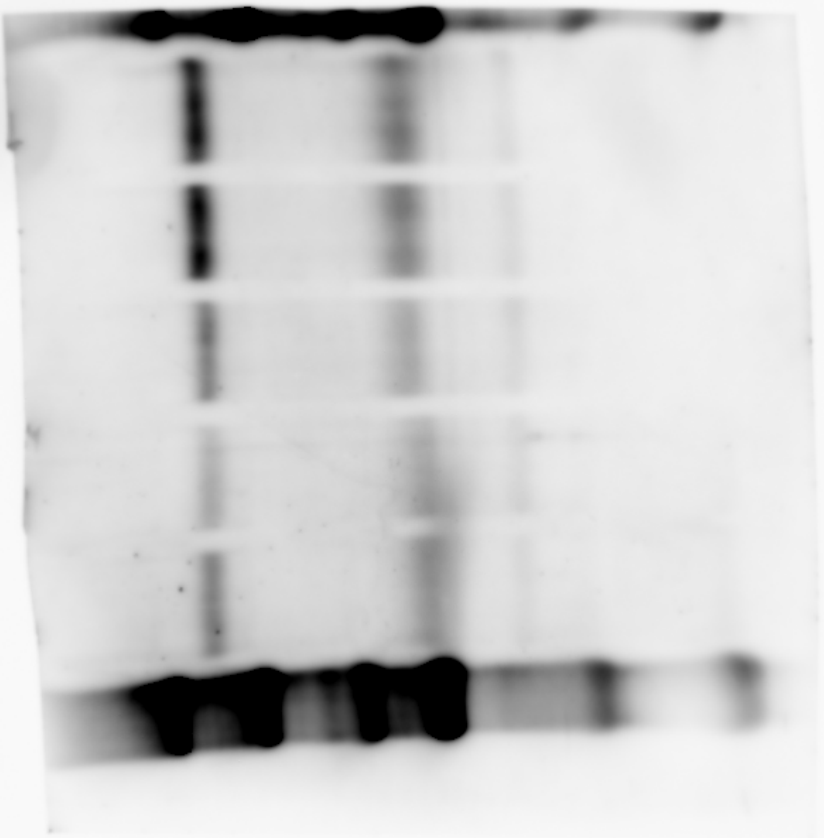

Supplementary Figure 1A

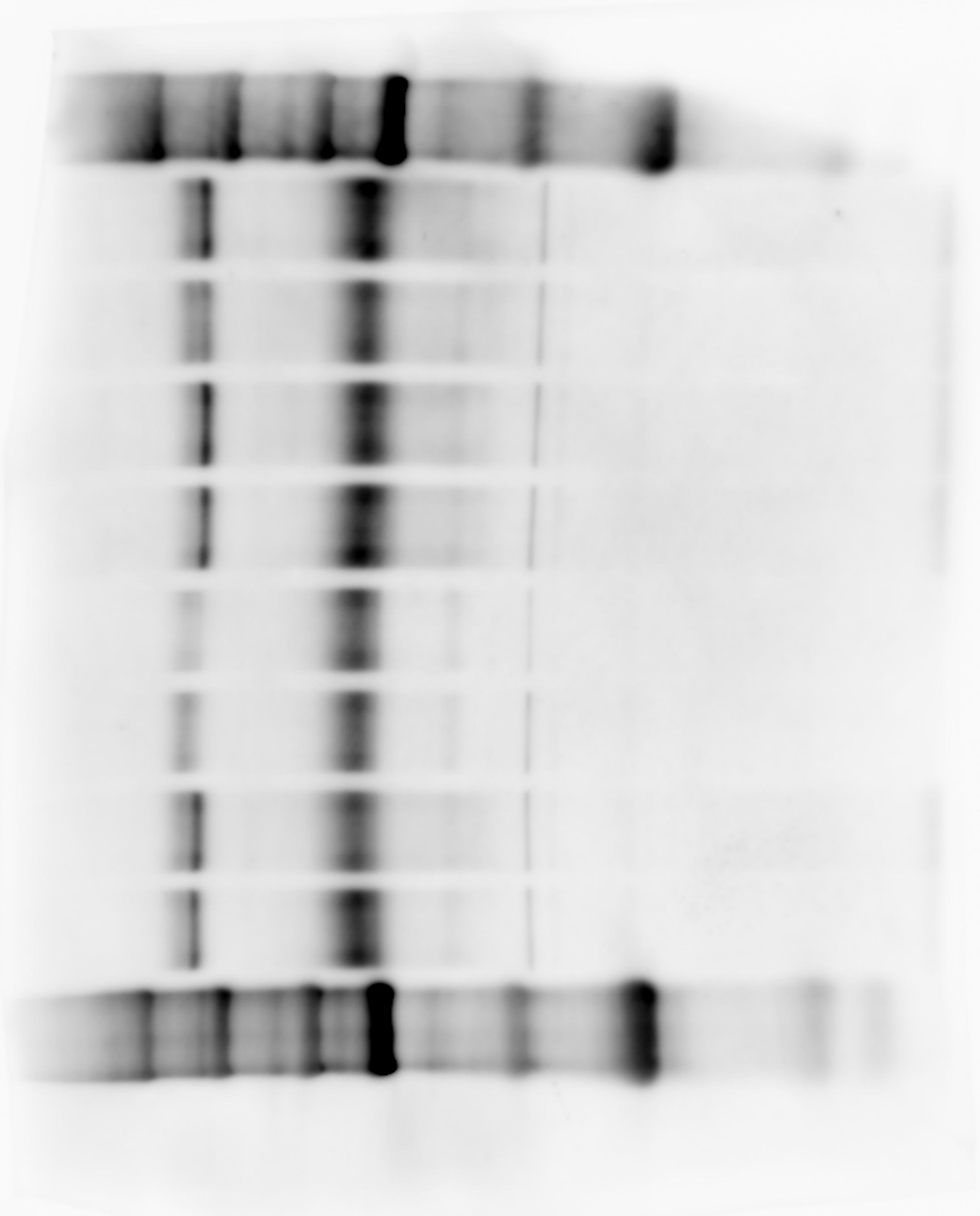

Supplementary Figure 1B

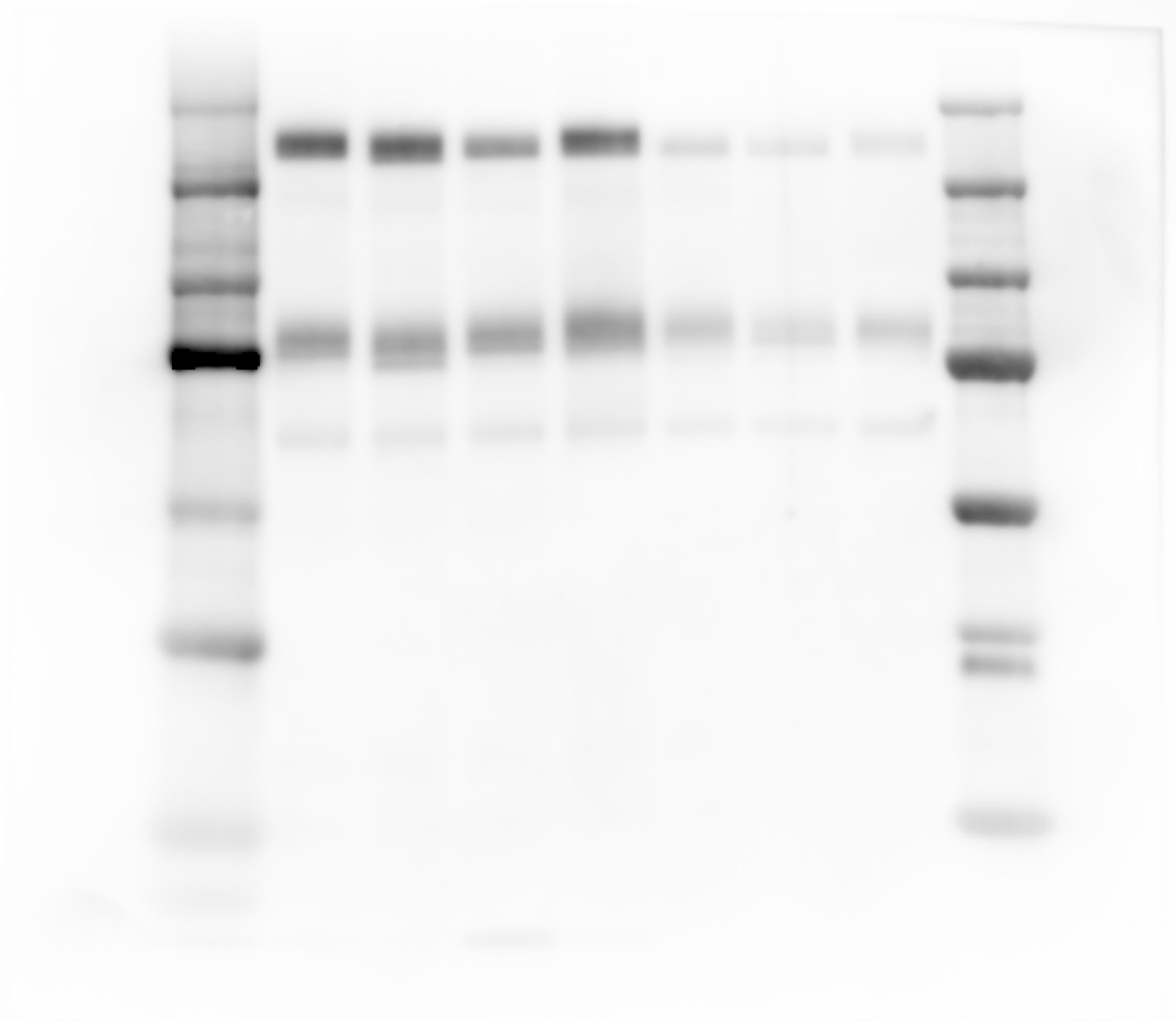

Supplementary Figure 1C

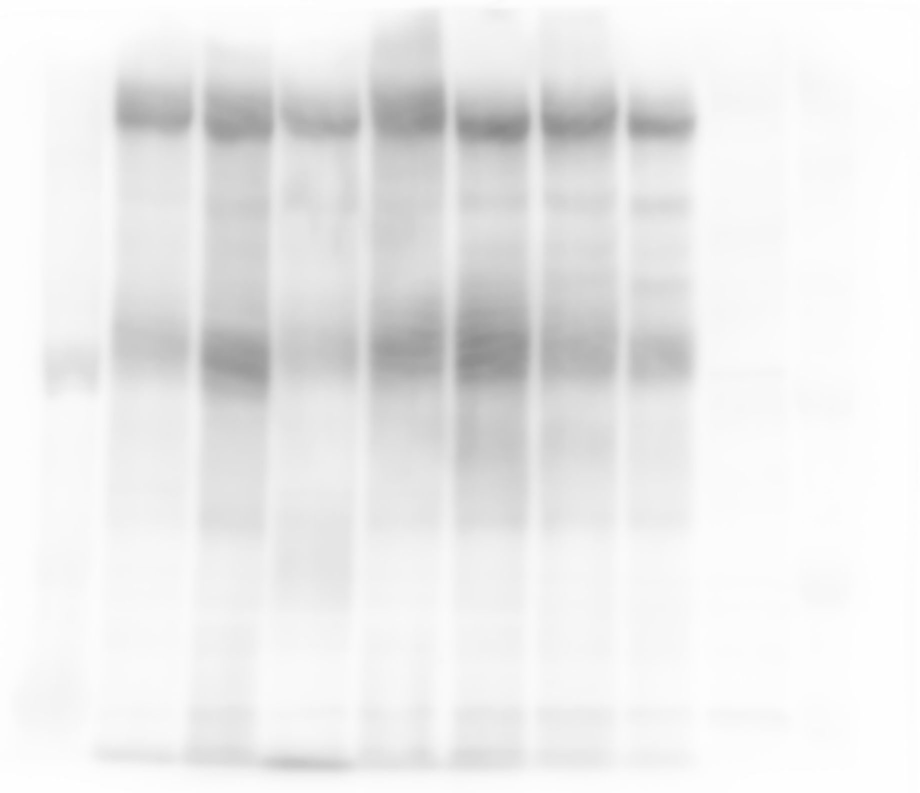

Supplementary Figure 1D
